# Genotyping common and rare variation using overlapping pool sequencing

**DOI:** 10.1186/1471-2105-12-S6-S2

**Published:** 2011-07-28

**Authors:** Dan He, Noah Zaitlen, Bogdan Pasaniuc, Eleazar Eskin, Eran Halperin

**Affiliations:** 1 Department of Computer Science, University of California, Los Angeles, CA 90095, USA; 2 Department of Epidemiology, Harvard School of Public Health, Boston, Harvard University, MA 02115, USA; 3 The Blavatnik School of Computer Science, and the Molecular Microbiology and Biotechnology Department, Tel-Aviv University, Tel-Aviv, 69978, Israel; 4 International Computer Science Institute, 1947 Center St., Berkeley, AC 94704, USA; 5 Program in Medical and Population Genetics, Broad Institute, Cambridge, MA 02142, USA

## Abstract

**Background:**

Recent advances in sequencing technologies set the stage for large, population based studies, in which the ANA or RNA of thousands of individuals will be sequenced. Currently, however, such studies are still infeasible using a straightforward sequencing approach; as a result, recently a few multiplexing schemes have been suggested, in which a small number of ANA pools are sequenced, and the results are then deconvoluted using compressed sensing or similar approaches. These methods, however, are limited to the detection of rare variants.

**Results:**

In this paper we provide a new algorithm for the deconvolution of DNA pools multiplexing schemes. The presented algorithm utilizes a likelihood model and linear programming. The approach allows for the addition of external data, particularly imputation data, resulting in a flexible environment that is suitable for different applications.

**Conclusions:**

Particularly, we demonstrate that both low and high allele frequency SNPs can be accurately genotyped when the DNA pooling scheme is performed in conjunction with microarray genotyping and imputation. Additionally, we demonstrate the use of our framework for the detection of cancer fusion genes from RNA sequences.

## Background

Recent advances in sequencing technologies have drastically reduced the cost of nucleotide sequencing [1,2] and are rapidly establishing themselves as very powerful tools for quantifying a growing list of cellular properties that include sequence variation, RNA expression levels, protein-DNA/RNA interaction sites, and chromatin methylation [3-8]. An expensive step in the sequencing process is sample preparation where time consuming procedures such as library preparation must be applied to each individual sample. This greatly reduces the utility of a sequencer for sequencing a small genomic region in many individuals because the cost of preparing each sample counteracts the efficiency of the sequencer. In fact the sequencing capacity in terms of the number of reads generated by the sequencer is often much higher than is necessary for the application. This raises the need for the development of multiplexing strategies that allow the processing of multiple samples per single sample preparation step at the cost of requiring additional sequencing capacity. However, in several practical scenarios, the overall cost can be reduced. One such multiplexing scheme is the use of overlapping pools [9-11]. In this scheme subsets of samples are mixed together into pools followed by a single sample preparation for each pool. Typically in such a sample preparation, a barcoding technique is applied so each read generated from the pool will be able to be identified as originating from the pool. By combining the results of the sequencing with the information on which samples appeared in which pool, the mixed information from each pool can be “decoded” to obtain information on the sequence of each sample.

Multiplexing pools are practical for sequencing a short genomic region in many individuals. As sequence capacity increases, it is likely that this technique will become even more practical in the future. Sequencing capacity is constantly increasing and therefore it is plausible that multiplexing pools will benefit whole-genome sequencing in the future. We note that in Erlich et al [9] the use of multiplexing has been proven in the lab, showing that this methodology is not merely a theoretical exercise. The current techniques for overlapping pool sequencing [9-11] are based on group testing or compressed sensing schemes. Their main limitation is that they are only applicable to detect rare variants. If a variant is common in the population, it will be present in almost every pool, causing the above pooling schemes to fail in identifying which subset of the samples contain the common variant.

In this paper, we present an alternate scheme for sequencing using overlapping pools which, unlike all previous approaches, is able to quantify both rare and common variation. The key idea underlying our scheme is that we formulate the pooling problem within a likelihood framework that provides several advantages over previous methods. Our scheme is flexible and can be applied to a wide variety of applications. We demonstrate this by applying the scheme to two very different applications, each of which takes advantage of the likelihood framework within our approach and is difficult to solve using previously proposed combinatorial methods.

The first application we consider is obtaining highly accurate genotype information for a set of individuals. Currently, genotype microarrays are the most accurate method for measuring individual genetic variation at a base-pair level at variable locations across the genome (Single Nucleotide Polymorphisms: SNPs). A typical array will collect up to 900,000 or more genotype calls at common SNPs across the genome. Using imputation techniques and a reference dataset such as the HapMap [12] or the 1,000 Genomes project, we can make predictions for the remaining common variants in the genome. While error rates of genotyping are usually less than .5% errors at imputed variants range from around 5% in Europeans, and it could be as high as 10-15% for non-European populations [12-14]. Imputation accuracy is particularly poor for rare SNPs and for SNPs in regions of low linkage disequilibrium. We introduce here a scheme for obtaining highly accurate genotype information on both common and rare SNPs by combining genotyping microarrays, imputation and sequencing in pools of samples. This application is possible because our likelihood framework allows us to integrate the information from the imputation into the procedure to help us “decode” the information obtained from each pool. Furthermore, our scheme allows us to utilize the variant frequency information obtained in each pool. Our results show that our algorithm is capable of calling rare SNPs with high accuracy, but in contrast to previous multiplexing methods, it can also call common SNPs with high accuracy, by combining the imputation data with the pooling scheme. In fact, the same experiment which can obtain genotype information for rare variants combined with imputation can obtain genotype information at the common variants. Importantly, the outcome of our approach results in genotype information on the common variation which is more accurate than what is collected using microarrays. This application is particularly practical because much of the follow up sequencing of populations will be done in the same cohorts in which genome-wide association studies were performed. For these individuals, genotyping at common SNPs using microarrays has already been performed and for many of these studies only the regions of interest are targeted for sequencing, or exome sequencing is being performed, which makes multiplexing pools a practical approach at present.

The second application we consider in this work is to rapidly screen for fusion genes in cancer samples. Fusion genes play an important role in cancers and are caused by genomic rearrangements in a tumor that create new genes consisting of several exons from one gene followed by several exons from a second gene. Our application considers the sequencing of RNA obtained from cancer tumors with paired-end reads. The read pairs of interest are ones that span exon boundaries with each read of the pair coming from a different exon. The majority of such read pairs will map to the same gene when aligned to the reference genome and. However, read pairs from fusion genes will map to two exons from different genes. One potential approach in identifying fusion genes is to search for read pairs that contain reads mapping to different genes. The main drawback of this approach is that it leads to a very high level of false positives making it difficult to distinguish actual fusions from experimental artifacts. Our application will mix RNA from a large number of cancer tumors into overlapping pools and utilize the likelihood framework to decode which fusion genes come from which samples. In order to accomplish that, we extended the basic overlapping pool model to consider different levels of expression for each gene. This can be estimated from the data. Our decoding scheme is based on a likelihood formulation which presents novel computational challenges compared to previous approaches. Each possible configuration (genotype assignment or gene-fusion assignment) is assigned a likelihood and the goal of the algorithm is to identify the most likely decoding. We identify good solutions for the problem by formulating a related problem as a linear program which we can efficiently solve. We note that these results are just the first steps in applying this framework to multiplexing sequencing pools and it is likely that better optimization algorithms and better designs of pooling schemes can lead to more substantial savings.

## Results and Discussion

### Genotyping using overlapping pools and imputation

We first report the results of applying our approach to genotyping individuals to obtain both common and rare variation using combining overlapping sequencing pools with genotyping and imputation. In this set of experiments, we utilize the 1958 Birth Cohort from the Wellcome Trust Case Control Consortium [15] data which contains approximately 1,500 individuals. These individuals were genotyped at approximately 500,000 SNPs. For every 10th SNP, we set the values of the genotypes to missing and applied MACH [16] using the HapMap data [17], an imputation algorithm, on these SNPs to make predictions. Since the SNPs were genotyped in the dataset, we can evaluate the accuracy of the imputation. We filter out any SNP with minor allele frequency lower than 5% since rare variants are easily genotyped using overlapping sequencing pools and the goal of these experiments is to evaluate the methods ability to genotype common variants. We simulate applying our method by generating sequencing reads by generating reads consistent with the true values of the genotypes at the missing SNPs for each pool and then apply our method to make predictions of the genotypes incorporating the imputation information. We then measure the increase in accuracy of our prediction relative to the imputation information.

For our experiments we consider a total of 100 individuals mixed into 36 pools which is a reduction of the total number of sample preparations necessary by 1/3. We use a very high coverage of 150 per individual within a pool for our experiments under the assumption that the bottleneck is not the coverage, but the number of pools, each which requires a single sample preparation step. We assume a sequencing error of 0.005. We measure the accuracy of the predictions by comparing the predicted genotypes to the true genotypes and only call a prediction correct if the genotypes are correct for all 100 individuals. We note that this is a very high standard and only 1 of our 100 SNPs have a correct imputation prediction. Our method has very high accuracy significantly improving over imputation. Table 1 summarizes the results.

**Table 1:** Results of genotyping using overlapping sequence pools with imputation information

Parameter Values Num Pools	Individuals = 100	
	Imputation Accuracy	LP Accuracy
36	0.01	0.98
30	0.01	0.87

### Genotyping using overlapping pools without imputation

We also apply our scheme to predict the genotypes without the imputation for rare variants such as those not found in the reference. The only difference in our methodology is that in the optimization problem, for the imputation vector we use a zero vector since we expect most individuals will not have the variant. This problem is actually much easier than the case of common alleles and we get perfect accuracy for the parameters above.

### Cancer fusion gene detection

We evaluate our approaches ability to detect cancer fusion genes using a similar simulation framework. In this application, RNA from different tumors is is mixed into overlapping pools and sequenced. In each pool we search for reads which cross exon boundaries from different genes and are evidence of fusion genes. Counts of these fusion genes in each pool are then decoded to identify the samples which contain the fusion genes.

We simulate this process by generating reads in a similar fashion to the genotyping without imputation simulations. We assume that we have 100 cancer samples where either 1, 2 or 3 of the samples contain a specific fusion gene. We assume a sequence error rate of 1% and vary the coverage and number of pools in our experiments. A difficulty in this application is that each individual has a different level of expression for each gene. We simulate this by randomly selecting an expression level in the range such that the concentration of the fusion gene in the RNA will differ by up to a factor of 10. Table 2 shows the results of our cancer fusion gene detection simulation experiment. For each experiment, we report the fraction of the time that the algorithm identified correctly which samples contain the fusion gene.

**Table 2:** Results of cancer fusion gene detection simulations

Parameter Values (Num Pools, Coverage, Error Rate)	# of Samples with Fusion		
	1	2	3
(10, 4 , 0.01)	0.980	0.760	0.340
(10, 12 , 0.01)	0.990	0.970	0.700
(10, 16, 0.01)	1.000	0.980	0.780
(10, 20, 0.01)	1.000	0.930	0.790
(10, 24, 0.01)	1.000	0.990	0.810
(10, 28, 0.01)	0.990	0.970	0.840
(4, 28, 0.01)	0.180	0.030	0.000
(6, 28, 0.01)	0.550	0.230	0.050
(8, 28, 0.01)	1.000	0.900	0.410

Each entry in the table is the fraction that the algorithm correctly identified the samples harboring the fusion gene.

## Conclusions

In this paper we have described a flexible framework for overlapping pool sequencing and two applications of this framework. We presented some results showing the bounds on the performance of decoding rare and common variants (in Methods). We argue that due to information theoretic bounds, common variants are impossible to decode without the addition of external information; we propose to use imputation results as possible external information. In practice, it is often the case that such information is given, as many of the samples have already been genotyped through the massive effort of genome-wide association studies; in addition, current cost of genotyping has reduced considerably and is negligible compared to sequencing costs (especially when considering gene-targeted sequencing).

Our decoding framework is likelihood based framework and is general enough to account for different types of data and error models. Particularly, we demonstrate how our method can be extended to the case where there are different unknown concentrations of each variant in each sample as motivated by the cancer fusion gene detection example. We note that our approach to detect fusion genes using RNA sequences can only detect fusion genes that are expressed in the tumor since we are sequencing RNA, but this is the case for a significant subset of the total fusion genes [18].

We expect that with improved optimization algorithms and better designs of pooling schemes, we can achieve even more substantial savings.

## Methods

We consider the scenario in which a set of *N* individuals are to be sequenced for any application such as a disease association study, or fusion-gene detection. The most straightforward approach would be to barcode the individual and sequence them separately. When *N* is large, or when the desired coverage is high, this approach is infeasible due to budget constraints. A few methods have been suggested to tackle this problem using a set of overlapping pools [9-11]. These methods are based on the following generic idea. Let the sequences of the individuals be represented by a matrix *G* = {*g_ij_*} of dimension *N* × *m*, where *m* is the length of the genome. *g_ij_* ∊ {0,1,2} is the number of occurrences of a genetic variant in position *j* of individual *i -* such variant could be a single nucleotide polymorphism (SNP), copy number variant (CNV), or a gene-fusion, as discussed in the introduction. The pooling based approach considers a {0, 1} matrix *A* of dimension *T* × *N*, representing a set of pools. Each row of *A* corresponds to a DNA pool; individual *j* participates in the *i —th* row if and only if *A_ij_* = 1. Thus, the matrix *A* provides a compact description of the study design. When the study is performed, under an error-free model, the pooling results are given as *Y* = *AG.* In principle, one can now decode the matrix by finding a solution to the set of equations *AX* = *Y.* In reality though the pooling results are not as accurate, and therefore current methods are using a rounded version of *Y;* for every *i*,*j*, we define *c_ij_* = 1 if *y_ij_* > 0 and *c_ij_* = 0 otherwise. Thus, if we replace the SUM operation by an OR operation then *AG* = *C.* Using this information, Erlich et al. [9],Prabhu et al. [10] and Shental et al. [11] provide a decoding algorithm which finds which individuals have *g*_ij_ > 0. For the simplicity of the exposition, we will assume from now on that only one variant is considered, and so *Y*, *C*, and *G* are column vectors of length *N.*

### A lower bound on decoding accuracy

Unfortunately, by collapsing the data to a {0,1} matrix resolution is lost, and therefore there is no hope in decoding all genetic variants from the pools if the number of pools is not large. Note that for a given variant, there are *3^N^* possible genotype vectors. The number of possible column vectors *C_j_* is 2*^T^.* Therefore, in order to be able to decode all individual genotypes we need *2^T^* > 3^N^, or *T* >*N.* Even without rounding, the number of possible vectors *B_j_* is at most (*2N*)*^T^*, and therefore even in the error-free case we need (*2N*)*^T^* > 3^N^, or . In practice, the pooling decoding methods work well when the allele frequency is low, under an error-free model. For a variant of allele frequency α, the number of possible genotype vectors is , and therefore, we get that in the case where the rounded solutions are provided (the matrix C), we need (2*N*)^*T*^ ≥ 3^*N*^, or , or *T* ≥ *—Nαlogα*, and if we are using the full information given by the matrix *B*, we need . Note that these are lower bounds, and it may theoretically be the case that a larger number of pools is required; however, it is easy to see that if *A* is chosen as a random matrix where each entry is 1 with probability 0.5, then the bounds given here are tight up to a constant factor (the proof is omitted from this version). Moreover, in [9-11], it is shown that using the matrix *C* one can decode low allele frequencies  then *T* = *O*(*logN*) suffices, which is consistent with the bounds we provide here. Since a random matrix provides a good decoding scheme in theory, we followed this intuition and generated a matrix *A* so that half of the entries in each row is 1 and the other half is 0. To obtain a better design matrix, we use local search; we repeatedly permute a random row and a random column and check to see if the Hamming distance between the permuted row/column and the other rows increased. If so, we keep the change, otherwise, we revert. After performing 1000 permutations, we result in a matrix whose rows and columns are farther apart, which improves our ability to decode.

### Incorporating imputation into decoding

As described above, from an information theoretical point of view, decoding the genotype vector is only possible when the allele frequency is low and therefore the genotype vector is sparse. For this reason, both Erlich et al. [9] and Shental et al. [11] make the connection between decoding and compressed sensing [19], where the requirement for the decoding success is based on the fact that the desired vector is sparse. We therefore suggest to incorporate imputation results into the decoding scheme; this allows us to overcome the information theoretical bound for the following reason. We can represent the true genotypes *G* as a sum of the (rounded) imputation predictions I, *i_ij_* ∊ {0, 1, 2}, and a set of imputation residual errors *R*, *r_ij_* ∊ {-2,1, 0, 1, 2}, where *G* = *I* + *R.* Then, the observed data can be represented as the pools’ results *Y* = *AG*, which is *Y* = *AI* + *AR.* Now, note that *R* is a sparse vector, and *I* is known; therefore, from a theoretical point of view, the above information theoretic lower bound does not hold on our case and there may be an algorithm that is able to decode the genotypes based on the sequencing and the imputation. In principle, we can solve for the imputation residual errors by solving the set of equations for *AX* = *Y - AI.* Once we obtain the residual vector, we can obtain the actual genotypes. In practice, as described below, we use the imputation dosage so our algorithm theoretically searches over the entire space, and not only over sparse vectors, but the search is pruned for vectors that are dense based on a likelihood model. As we show in the results section, this yields an improved imputation accuracy for high allele frequency SNPs.

### Pooling using read counts

Our approach differs from previous approaches in that we are considering the matrix *Y* and not *C.* As discussed above, this should allow us a gain of approximately log *N* factor in the number of pools needed, at least for higher allele frequencies. However, in order to do so, we need to explicitly model the sequencing errors. The error model may be different, depending on the application at hand. We will describe here the model we use for the detection of mutations (SNP calling). There are three main sources of noise that we include in the model:

1. There are slight differences in the concentration of each individual’s DNA in each pool. This *pooling noise* is modeled as a normally distributed noise added to each non-zero element of A with mean 0 and variance *σ_p_.* Thus, we set

2. There is a variance in the coverage of any specific region in the genome. We denote by *L* the length of the sequenced genomic region; if the total number of bases sequenced is *λLN*, then we expect that each base will be covered by λ reads on average. λ is often termed the expected *coverage.* We will denote the number of reads covering individual *i* by *r_i_*_1_, *r_i_*_2_ (corresponding to the two chromosomal copies). Then, *r_ij_* is Poisson distributed, with mean m_i_. Prabhu et al. [10] showed that the m_i_ are approximately drawn from a Gamma distribution with *α* = 6.3 and *β* = λ/*α* for Illumina Solexa sequencers. We note that for a given variant it is easy to infer the value of *m_i_*, since it is shared across all individuals in all pools. Thus, we have

*m*_i_ ~ Γ(*α*, *β*), *r*_ij_ ~ *Poisson*(*m_i_*)

3. The third source of error is sequencing error. The sequencing error rate depends on the location of the base in the read, but since the location of the base is uniformly distributed, we simply model the error rate by a constant probability *ε* for a substitution (1% is an acceptable estimate).

The above procedure produces a matrix *Â* of noisy pools and a pair of vectors  of noisy sequence reads; the number of sequence reads  is generated by a Poisson distribution with an expectation that depends on the genotype *g_i_*, and the coverage *m_i_*, followed by a Binomial distribution to model the errors as explained above. *R*^0^ corresponds to the reads with the major allele, while *R*^1^ corresponds to the reads with the minor alleles. Note that even if *g_i_* = 0, if *ε* > 0, then expected number of reads with the minor allele will be greater than 0 because of errors. The pooling results are given by (*Y*^0^, *Y*^1^), where *Y^k^* = *ÂR^k^.*

### A likelihood model

Given the pooling results *Y*, we need to find a decoding algorithm that estimates *G* from *Y.* To do so, we define a likelihood model which can evaluate each putative solution. Our likelihood model takes into account both the error model, as well as population genetics data and external information when available. We decompose the likelihood *L*(*G; Pools*) into several functions, and take their product as a composite likelihood.

#### Hardy-Weinberg Equilibrium

We first note that the overall allele frequency  of the SNP can be estimated as the average across all pools. We can now compute the Hardy-Weinberg (HW) probability of the observed genotypes , where *n*_0_, *n*_1_, *n*_2_ are the genotype counts in *G.* Using Bayes law we have

Assuming no prior information, we observe that maximizing *Pr^HW^*(*Pools* | *G*) is equivalent to maximizing *Pr^HW^*(*G* | *Pools*)*.* We denote .

#### Likelihood of the observed reads

We compute the probability of the observed reads in the pools given G based on the noise model. Note that the only unknown in the noise model is the concentrations of the individuals in the different pools. This is true since the coverage in any given region can be easily estimated. Assume that λ is the coverage. Then, the number of reads with the minor allele (or major) contributed by individual *j* in pool *i* are Poisson distributed with *Â_ij_λG_j_* (or *Â_ij_λ*(2 *- G_j_*))*.* Since the sum of Poisson distributions is Poisson distributed, we have that  is Poisson distributed with a known expectation and thus we can write its likelihood. We denote this function by *f^n^*^oi^*^se^*(*G*)*.* In order to find the concentration values *Â*_i_*_j_* we need to use external information. One such possibility could be to genotype small set of SNPs across the population and use those as the ground truth in order to tune the values of *Â_ij_.* These SNPs provide a set of linear equations for the values *Â;* for each pool we have one equation per SNP, and the number of variables is *N.* Therefore, genotyping as many as *O*(*N*) SNPs and using a least squares approach guarantees an accurate estimate of *Â_ij_.*

#### Likelihood of imputed data

Due to the bounds given on the possibility for detection, it is clear that without external information we will not be able to do much better than detecting rare SNPs. One natural choice for external data could be the genotypes of the individuals using microarrays. Today’s genotyping technology is extremely cheap compared to sequencing, and the genotyping of thousands of individuals is feasible within a given study. The genotype information, however, provides the information about less than a million SNPs and another million CNVs across the genomes, while many other genetic variants are left unmeasured. To cope with this, imputation methods have been developed, in which nearby SNPs are used to impute unmeasured variants using the linkage disequilibrium structure of the genome [16,20]. However, this process is inevitably noisy, especially when imputing SNPs of low allele frequencies or SNPs in regions of low linkage disequilibrium. Together with the pooling information we are able to provide a much more accurate calling of the imputed SNPs in all ranges of allele frequencies and linkage disequilibrium patterns. The output of the imputation method typically provides a distribution of the possible genotypes. For each individual *j*, we can assume that there is a given probability *h*_i_(0), *h_i_*(1), *h*_i_(2), where *h_i_*(*j*) is the probability that individual *i* has *G_i_* = *j.* We can now use the imputation results for our likelihood model, by writing .

### A decoding algorithm using linear programming

We use a linear program to bound the possible errors of each of the pools. If the coverage for the SNP is λ, we have that the pools should roughly satisfy λ*ÂG* = *Y.* We can therefore solve the following linear program:

The linear program provides a lower bound on the best possible *l*_1_ distance between λ*ÂG* and *Y* as well as returning a solution *G* which is close to the imputation prediction *I. β* is a parameter that trades off the relative importance of being close to the imputation vector compared to being consistent with the pools. Note that if *Y_j_* is distributed as Poisson with expectation *µ_j_* for which *Y_j_ - µ_j_* = *x_j_*, then

Therefore, we get that .

### Application to gene fusion detection

In order to detect gene fusions, we make several changes and extensions to the model presented above. The major additional complication in detection of fusion genes is that each sample may have a different expression level for a particular fusion gene. Even if we include the same amount of RNA from each tumor into each pool, the relative concentration of each gene will differ in each sample. However, this concentration is approximately constant across pools. Let *e_ij_* be the normalized expression level of a particular variant *j* (in this case a fusion gene). Whether or not an individual *i* has the variant *j* is encoded as *G* = {*g_ij_*}, *g_ij_* ∊ {0, 1}. We define the matrix *H* = {*e_ij_g_ij_*} as the concentrations of the samples and the results of the pools (assuming no noise) will then be *Y* = *AH* instead of *Y* = *AG* as in the genotyping application. In this application, we can also assume that the matrix *G* is sparse, but in order to perform the decoding, we must also estimate *e_ij_* for the non-zero values of *g_ij_*.

It is possible to estimate *e_ij_* because they are constant across pools, however this introduces additional complexities in the optimization. We take advantage that fusion genes are very rare and most fusion genes are not shared across tumors. We constrain our optimization to allow for a maximum of *k* tumors to contain a given sample. We note that we only need to estimate the values of *e_ij_* corresponding to non-zero elements of *g_ij_*. To perform the optimization we enumerate over all  possible genotype vectors and for each vector we estimate the corresponding *e_ij_* values.

Since optimizing the likelihood function for each possible genotype vector is computationally impractical, we solve a linear program as a method to quickly eliminate poor solutions. Let A* be a matrix consisting of the only the columns of *A* corresponding to the non-zero entries of the genotype vector. If *x* is a vector which has a length the same as the number of non-zero elements in the genotype vector, the solution to *A* * *x* = *Y* will be an approximate estimate of the values for *e_ij_*. We can incorporate errors by adding a vector of all 1s to A* and appending a term to x corresponding to the amount of errors expected in each pool. For the top 100 estimates obtained by using the pseudo-inverse, we then perform a grid search over the values of *e_ij_* using the likelihood function described above.

## Competing interests

The authors declare that they have no competing interests.

## Authors' contributions

D.H., N.Z., B.P., E.E. and E.H. developed the method. D.H., N.Z., B.P. performed the experiments. D.H., E.E. and E.H. wrote the manuscript.
